# Enhancing Diabetes Self-Management Through Collection and Visualization of Data From Multiple Mobile Health Technologies: Protocol for a Development and Feasibility Trial

**DOI:** 10.2196/13517

**Published:** 2019-06-03

**Authors:** Ryan J Shaw, Angel Barnes, Dori Steinberg, Jacqueline Vaughn, Anna Diane, Erica Levine, Allison Vorderstrasse, Matthew J Crowley, Eleanor Wood, Daniel Hatch, Allison Lewinski, Meilin Jiang, Janee Stevenson, Qing Yang

**Affiliations:** 1 Duke University School of Nursing Durham, NC United States; 2 Center for Applied Genomics & Precision Medicine Duke University School of Medicine Durham, NC United States; 3 New York University Rory Meyers College of Nursing New York, NY United States; 4 Center of Innovation to Accelerate Discovery and Practice Transformation Durham Veterans Affairs Medical Center Durham, NC United States; 5 Division of Endocrinology, Diabetes and Metabolism Duke University School of Medicine Durham, NC United States; 6 Pratt School of Engineering Duke University Durham, NC United States; 7 Duke University School of Medicine Durham, NC United States

**Keywords:** self-management, technology, type 2 diabetes

## Abstract

**Background:**

Self-management is integral for control of type 2 diabetes mellitus (T2DM). Patient self-management is improved when they receive real-time information on their health status and behaviors and ongoing facilitation from health professionals. However, timely information for these behaviors is notably absent in the health care system. Providing real-time data could help improve patient understanding of the dynamics of their illness and assist clinicians in developing targeted approaches to improve health outcomes and in delivering personalized care when and where it is most needed. Mobile technologies (eg, wearables, apps, and connected scales) have the potential to make these patient-provider interactions a reality. What strategies might best help patients overcome self-management challenges using self-generated diabetes-related data? How might clinicians effectively guide patient self-management with the advantage of real-time data?

**Objective:**

This study aims to describe the protocol for an ongoing study (June 2016-May 2019) that examines trajectories of symptoms, health behaviors, and associated challenges among individuals with T2DM utilizing multiple mobile technologies, including a wireless body scale, wireless glucometer, and a wrist-worn accelerometer over a 6-month period.

**Methods:**

We are conducting an explanatory sequential mixed methods study of 60 patients with T2DM recruited from a primary care clinic. Patients were asked to track relevant clinical data for 6 months using a wireless body scale, wireless glucometer, a wrist-worn accelerometer, and a medication adherence text message (short message service, SMS) survey. Data generated from the devices were then analyzed and visualized. A subset of patients is currently being interviewed to discuss their challenges and successes in diabetes self-management, and they are being shown visualizations of their own data. Following the data collection period, we will conduct interviews with study clinicians to explore ways in which they might collaborate with patients.

**Results:**

This study has received regulatory approval. Patient enrollment ongoing with a sample size of 60 patients is complete, and up to 20 clinicians will be enrolled. At the patient level, data collection is complete, but data analysis is pending. At the clinician level, data collection is currently ongoing.

**Conclusions:**

This study seeks to expand the use of mobile technologies to generate real-time data to enhance self-management strategies. It also seeks to obtain both patient and provider perspectives on using real-time data to develop algorithms for software that will facilitate real-time self-management strategies. We expect that the findings of this study will offer important insight into how to support patients and providers using real-time data to manage a complex chronic illness.

**International Registered Report Identifier (IRRID):**

DERR1-10.2196/13517

## Introduction

### Background

As most of diabetes care occurs in outpatient settings and involves ongoing patient self-management, mobile health (mHealth) technologies may greatly improve diabetes management and health outcomes. mHealth involves the use of mobile devices to support continuous health monitoring and healthy behaviors [1]. Mobile devices include mobile phones and sensors that are worn, carried, placed in the physical environment, or accessed by individuals during normal daily activities [2]. These devices allow reporting of patient data such as blood glucose through a wireless glucometer, weight through a cellular-enabled scale, and physical activity through a wireless accelerometer in near real-time in the patient’s daily environment. Moreover, these data can be transmitted to clinicians and health systems and may lead to the development of precision health strategies [3].

According to the Pew Research center, more than 92% of US adults own a cell phone, and more than 77% own a smartphone [4]. Furthermore, 84% of low-income individuals in the United States now own a cell phone, and almost 70% own a smartphone. Low-income racial/ethnic minorities are actually more likely than low-income whites to own mobile devices and to use features such as SMS (short message service) text messaging or smartphone apps [4]. Research demonstrates that mHealth tools are useful for diabetes self-management [5,6] including collaborative decision making between providers and patients [7]. Thus, mHealth technologies have great potential to facilitate wide delivery of diabetes treatment and enhance the development of self-management tools for diverse populations.

The study protocol described in this manuscript extends current research by using multiple mHealth technologies to provide diabetes-related data to help patients and their clinicians better understand illness dynamics and develops personalized approaches through data visualization to improve health outcomes in type 2 diabetes mellitus (T2DM). Specifically, we describe methods for identifying precision health strategies to help patients self-manage using multiple types of self-generated diabetes-related data. We will also demonstrate how clinicians can help guide near real-time patient self-management by collecting and aggregating streams of health data from multiple mobile technologies and then creating a variety of data visualizations that we present to both participants and clinicians. Our goal is to understand what kinds of visualizations patients of varying backgrounds and clinicians need. Furthermore, we describe more about the need for understanding what kind of alerts patients might find useful to self-manage their diabetes. These alerts, or algorithms, will be developed from health data from the mobile technologies and associated electronic health record (EHR) data from patients.

### Aims

The aims of this project are to examine the feasibility and utility of having patients self-monitor multiple types of diabetes-related data (blood glucose, weight, physical activity, medication adherence) using mHealth technologies (wireless glucometer, cellular-enabled body scale, wrist-worn accelerometer, and medication adherence SMS text message surveys). This will allow us to examine trajectories of diabetes-related variables (blood glucose, weight, physical activity, medication adherence) and challenges in self-management that patients face at points in time. We are also exploring the challenges and successes of patients self-managing diabetes using mHealth technologies through interviews and data visualizations. And finally, we are exploring clinicians’ perspectives and input on using these data to develop algorithms for software that will facilitate patient self-monitoring and meet near real-time self-management needs. Results from this study will be used for the integration of data from mHealth tools into EHRs and for developing new models of care delivery to support diabetes management.

### Conceptual Framework

The study protocol is supported by the adaptive leadership framework [8], which divides health challenges into 2 types: technical and adaptive [9,10]. Technical challenges such as how and when to measure blood sugar and what the appropriate medication dosages are to manage diabetes are addressed by the clinician with technical solutions. Adaptive challenges require the patient to adjust to new conditions and do the work of learning and making behavioral changes, for example, incorporating exercise into everyday life. Adaptive leadership is the work that clinicians do to help patients perform this work [11]. Using multiple types of self-generated diabetes self-monitoring data, clinicians can support patients in near real time to respond to adaptive challenges.

**Figure 1:**
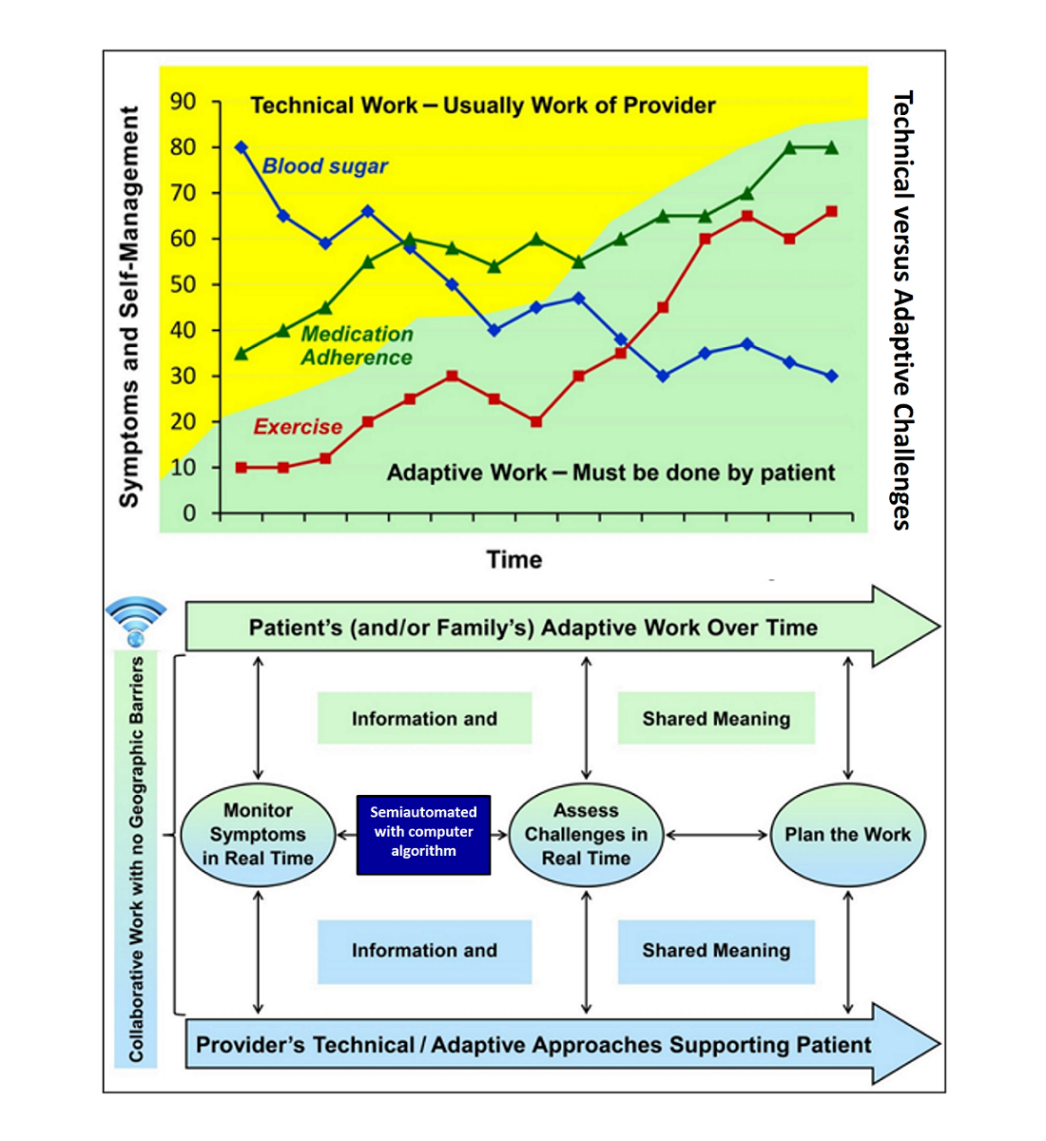
Mobile health in a collaborative work relationship.

Figure 1 (adapted from National Institute of Nursing Research 1P30NR014139-01) shows how mobile technologies might help patients monitor behavioral, symptom, and biophysical trajectories. These trajectories, shown in the top half of the model, suggest these variables are dynamic. For example, when patients are able to overcome challenges in scheduling exercise and medication adherence, these behaviors increase, and their blood sugars are likely to decrease. The goal of adaptive leadership is for clinicians to address technical challenges, such as finding the appropriate medications and dosages, and also to help patients address adaptive challenges such as lifestyle changes by facilitating their adaptive work for self-management. Over time, as patients address the challenges of self-management and their adaptive work increases, the amount of technical work needed by clinicians will decrease. The trajectories of the signs and symptoms of T2DM, such as high blood sugar, will also decrease. The lower half of the model illustrates that mobile technology can provide important information to be used in the collaborative work-relationship to facilitate both the adaptive work of patients and the technical and adaptive approaches used by clinicians to support patients. According to this framework, patients and clinicians collaborate to monitor symptom dynamics and self-management techniques. Together, they assess adaptive challenges and plan the technical and adaptive work needed to help patients meet their diabetes goals such as weight loss, medication adherence, and so forth. We considered this framework when designing the protocol described below including the questions we ask both patients and providers.

## Methods

### Study Phase 1: Software Development

**Figure 2:**
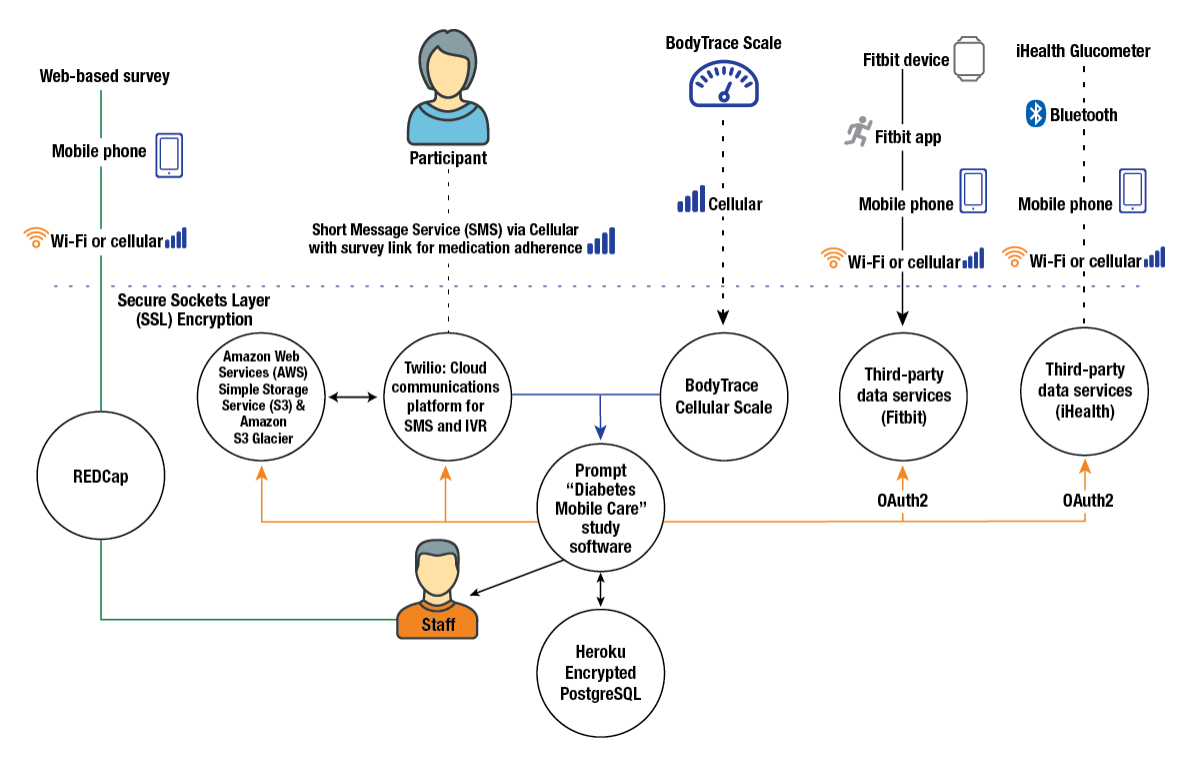
Data flow connecting participant device data to study software. IVR: interactive voice response.

We began with software development (Figure 2). For Phase 1, we selected consumer-friendly devices that patients could easily use for their diabetes self-management. We chose a wireless glucometer by iHealth, a wrist-worn accelerometer by Fitbit, and a cellular-enabled scale by BodyTrace. Using Prompt, a research platform designed to collect, analyze, and message patients about mHealth data [12], we programmed the ability to pull in data from these respective companies via their application programming interfaces (APIs). Every day, Prompt uses an authentication token to request data via each API for each participant in the study. Those data are collected over the course of the study and stored in a secure database. A study coordinator is also able to view data in aggregate that participants transmit over time. In addition, Prompt was programmed to send out a scheduled SMS text message with a survey link every 2 weeks. This link allowed participants to complete a short survey about their medication adherence via their phones in the Web-based Research Electronic Data Capture (REDCap) Web-based platform hosted at Duke University [13]. REDCap is a secure, Web-based application designed to support data capture for research studies, providing: 1) an intuitive interface for validated data entry; 2) audit trails for tracking data manipulation and export procedures; 3) automated export procedures for seamless data downloads to common statistical packages; and 4) procedures for importing data from external sources.

### Study Phase 2: Data Collection

Following software development, we received institutional review board (IRB) approval for phase 2. In this phase, we are conducting a mixed methods explanatory sequential designed study [14] for which we recruited 60 adults (aged ≥18 years) with T2DM. This is a 2-phase design where quantitative data are collected and followed by qualitative data collection. Patients were recruited from a primary care clinic associated with an academic medical center in the southeastern United States and through local advertisements. Eligible participants met the following inclusion criteria: (1) aged ≥18 years, (2) able to speak and read English, (3) diagnosed with T2DM, (4) told by their primary care provider to monitor their blood sugar, (5) own and use a smartphone, (6) capable of giving informed consent, and (7) able to travel for study enrollment. Participants were excluded if they had a preexisting severe medical condition(s) that would interfere with study participation (eg, renal failure, severe orthopedic conditions or joint replacement scheduled within six months, paralysis, or cancer). A proactive effort was made to enroll patients at various stages of diabetes mellitus, with treatment regimens including both oral and injectable medications were included. We purposefully targeted patients with a range of diabetes severity and included at least 25 patients with a hemoglobin A_1c_ (HbA_1c_)>7.5%. Patients were not required to have Wi-Fi or in-home internet; a smartphone with an internet connection was adequate. All other devices (eg, glucometer, cellular-enabled scale, and accelerometer) were provided to patients. Patients were excluded if they (1) had active dementia or psychiatric illness, (2) resided in a nursing home, or (3) were participating in another self-management study.

We used a study recruiter to identify patients by reviewing EHRs at primary care clinics. Eligible participants received a letter inviting them to participate. Those who were interested contacted the study recruiter and were screened over the phone. Baseline appointments were scheduled. We also used Web advertisements and posted flyers to recruit half of our participants from the local community.

#### Sample Size

Our goal is to obtain information critical to plan a larger trial and to understand how to use data from these trials in clinical practice. Accordingly, our sample size is based on our aims to determine feasibility and acceptability on using these devices and getting feedback from multiple stakeholders involved with their use, not on power to detect significant effects of self-monitoring on outcomes (eg, weight, HbA_1c_, and exercise). Our sample size of 60 patients is designed to provide a sufficient number of participants to obtain high retention rates, means, and variance estimates of end points that can be used to design and power the future trial. Our sample size of up to 20 clinician interviews is based upon estimates that we will likely reach data saturation within 12 or more interviews [15].

#### Baseline Appointment

To minimize participant burden, the baseline appointment occurred following a clinic visit or at a time deemed convenient for participants. During the in-person appointment, the recruiter (1) obtained signed informed consent, (2) administered surveys to record demographic factors, (3) measured patients’ health literacy using the Rapid Estimate of Adult Literacy in Medicine [16] and electronic health (eHealth) literacy on the use of information technology for health using the eHealth Literacy Scale [17], (4) evaluated patients’ perceived usefulness and ease of use of mobile technologies, (5) measured patients’ exercise frequency using the Godin Leisure-Time Exercise Questionnaire [18], and (6) collected data from patients’ EHRs (ie, HbA_1c_, height, weight, blood pressure, heart rate, and medications). We collected demographic data including race/ethnicity, marital status, income (categorical), educational attainment, employment, age, sex, and duration of disease. These data allowed us to evaluate differences in the data collected by characteristics or groups of patients and to perform subgroup analyses.

Study details and expectations were discussed during the appointment and included risks and costs that participants might incur from using cell phone data and SMS text messages. The study recruiter helped patients set up the mobile technologies, including 2 associated glucometer and accelerometer smartphone apps, and answered any questions. The research assistant also instructed patients how to monitor and record progress. If patients needed follow-up then a call was placed by the recruiter to participants to address questions about the devices and study procedures and to provide a reorientation if needed. Participants were given a study number to call if they had questions or problems with the study. A research assistant was available to participants throughout the study to provide technical support.

#### Daily Monitoring

Patient weight, physical activity, and blood glucose were self-monitored via devices provided at baseline (Table 1). Patients were asked to monitor weight daily using a cellular-enabled scale by BodyTrace that connected to a cellular network. Patients were advised to weigh daily because more frequent weighing promotes better weight outcomes than less frequent weighing [19,20]. Patients also received a Fitbit Alta, a reliable and validated triaxial accelerometer for exercise monitoring [21,22]. Participants were instructed to wear the Fitbit daily. The Fitbit tracked daily number of steps taken, distance traveled, and intensity of exercise and provided feedback on these data points to patients. The devices could be worn in the shower or while swimming and were to be worn 24 hours/day and removed only to recharge the battery once per week or when indicated. The Fitbit was tethered to the Fitbit app on the participants’ smartphone via Bluetooth.

Glucose readings were tracked using a Food and Drug Administration–approved glucometer by iHealth (model BG5). Participants were instructed to monitor glucose based on the recommendations from their doctor. Like the Fitbit, the glucometer was tethered to a companion smartphone app via Bluetooth, which acted as an automatic logbook to store readings, notes, and medication dosages. Patients were given a 6-month supply of test strips for the device.

In addition to data tracking on the devices, patients received an SMS text message every 2 weeks with a link to a survey on medication adherence (over the previous week) that they completed via their smartphone. Participants clicked on the survey link, which took them to a Web-based survey in a REDCap database. We used a 3-item measure of nonadherence by Voils et al [23]. Participants were asked if over the past 7 days they (1) took all doses of my diabetes medication, (2) missed or skipped at least one dose of my diabetes medication, and (3) were not able to take all of my medication. Response options ranged from *never* (0) to always (5; Cronbach alpha=.84) [23]. Response items were scored to assess the extent of nonadherence.

Those who failed to transmit data for a period of 7 days or longer were contacted over the phone by a research assistant to encourage reconnection with the study. If there was no response, the participant was contacted again via phone call or email 1 week later. Nonresponse at that time was considered elective withdrawal from the study. We attempted to contact any participants who withdrew to discuss their reasons for withdrawal. However, no more than 2 follow-up phone calls were made. We asked participants at baseline to please notify us of travel or other situations during which they were not able to transmit data. We made exceptions for participants who were on vacation or traveling. All mobile devices remained active until the end of the study to allow for reconnection at any point.

**Table 1:** Mobile devices and data collection

Data	Instrument	Description	Data points
Exercise	Triaxial accelerometer and associated fitness app by Fitbit	Tracks data on the frequency and timing of steps	Daily: steps, minutes sedentary, minutes active, distance traveled
Weight	Cellular-enabled Scale by BodyTrace	Tracks weight	Daily weight
Glucose	Food and Drug Association–approved wireless glucometer by iHealth	Tracks blood glucose readings	As prescribed by primary care physician
Medication adherence	Self-report via short message service text message [23]	Medication adherence over the last week	Baseline, biweekly up to 6 months
Hemoglobin A_1c_	Electronic health record (EHR) laboratory results	Average level of blood sugar over the previous 3 months	Gathered from the EHR as available at baseline and 3 and 6 months post baseline

#### Six Months Post Baseline

After 6 months, participants were sent a link via email to complete a follow-up survey on their experiences in the study; this was administered again using REDCap. Participants who completed the study and the final survey were able to keep the mobile devices.

### Study Phase 3: Data Visualizations and Interviews

#### Participant Interviews

We will complete semistructured telephone interviews with a subset of participants (n=20). To gain diverse perceptions, we will purposefully select participants for interviews based on frequency of data transmission (eg, consistent or inconsistent over the study period), management of diabetes (eg, controlled or not controlled), demographic characteristics (eg, age and race), and severity of diabetes (eg, A_1c_ level). All interviews will occur via telephone and will be conducted by 2 trained research assistants. The purpose of the interviews is to obtain participants’ perceptions of the study, use of the mobile devices in diabetes self-management, and effectiveness of the data visualizations. The topic of questions for participants include those about the usefulness of the mobile devices and their data, and if they helped with diabetes self-management, challenges. Furthermore, we will provide participants with data visualizations of their own data, and we will discuss how they could be curated to be useful.

#### Data Visualizations

**Figure 3:**
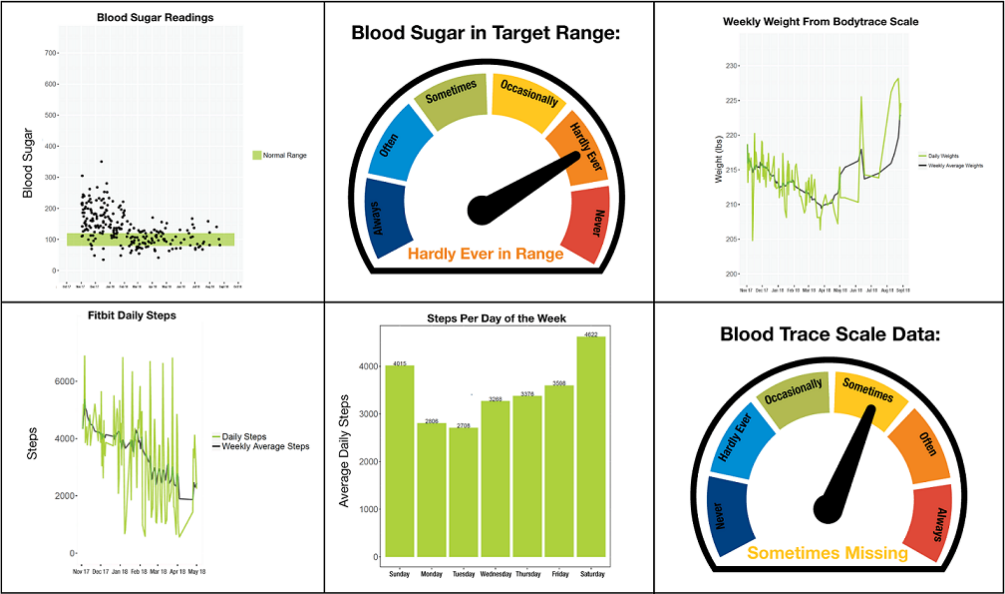
Example data visualizations from multiple mobile health technologies.

Before the interview, each participant will be sent a copy of their data visualizations via postal mail or email, depending on participant preference. These data visualizations will include graphic representations of the patient’s weight, blood glucose, and exercise as obtained from the devices (Figure 3). They will be plotted as trajectories that will allow us to conduct analyses and identify missing data points and trends that lead to attrition. The research assistant will use these data visualizations to facilitate discussion about the challenges patients face in self-management and the personalized practices patients use to succeed. Details on how we will approach the development of these visualizations are published elsewhere [24]. All interviews will be recorded, stored in an IRB-approved secure database, and transcribed verbatim.

#### Clinician Interviews

We will conduct interviews with health care clinicians following the completion of patient interviews. This will allow us to explore ways to address collaborative work in diabetes self-management using the device data that could be transmitted in near real time. We will obtain a convenience sample of up to 20 clinicians: 10 with prescribing privileges (eg, medical doctor, nurse practitioner, and physician assistant) and 10 case managers/nurses. This will provide us with diverse perspectives on how to use the device data in new care delivery models and in workflow integration. Questions will be similar to those asked of patients. All participants will provide written informed consent. Our team members will identify clinicians from primary care and endocrinology clinics who may be interested in participating. Study details and expectations will be discussed, questions about the study will be answered, and if interested, the study recruiter will schedule an appointment to participate.

We will present data trajectories from the patients [24] and interview themes from the patient participants. This will allow us to explore clinicians’ perspectives and input on using the device data to develop algorithms for software that will facilitate patient self-monitoring and provide feedback to clinicians within existing and future care delivery models.

### Changes to the Protocol

No changes to eligibility criteria or outcomes changes were made during the study. We did change clinician focus groups to interviews. Interviewing emerged as a better opportunity to discuss various data visualizations and to receive feedback about workflow integration among diverse clinicians

### Data Analyses

All statistics will be calculated using SAS Version 9.3 (SAS Institute Inc.). Baseline demographic and clinical characteristics of enrolled participants are summarized by mean and SD for continuous variables and frequency and percentage for categorical variables. A significance level of 0.05 is considered as statistically significant for all tests.

Using the data collected in Prompt, we will create data visualizations using 2 different data visualization software packages (Tableau and R) and pilot their use with participants via a phone interview. We will use the interviews as an iterative process to discover how to present the visualizations back to patients and to refine the visualizations. We will start with plotting the actual data transmitted from each of the devices from individuals over the entire follow-up time to show patients overall trend in blood glucose, exercise level, and weight. For blood glucose data, we will add a band of ideal blood glucose range to help patients visualize if their measurements are within targeted range. For Fitbit data, we will plot both the average active steps daily and weekly over time and also plot the steps by weekday to explore if there are any cyclic and long-term drift trends in their exercise level. For weight data, similarly, we will plot both daily weight and weekly average to show overall trend. Further details on this approach are described elsewhere [24].

#### Patterns of Missing Data and Attrition

The primary analytical aim of this study is to estimate individuals’ performance of all the tasks involved in the daily monitoring of weight, exercise, and glucose and responding to the SMS text message surveys for medication adherence. A proxy for this estimate will be the percentages of the 180 days of data points from the Fitbit, the wireless scale, and the glucometer that were transferred. We will calculate the percentage of 180 days on which each type of data was transferred for each participant. Patient’s respondence to SMS text message surveys to medication adherence will be calculated by the number of surveys received out of 13 time points (baseline and every other week for 6 months). To examine if the performance of all tasks differ by important clinical groups, including age (<50 vs ≥50 years), HbA_1c_ group, (<7 vs ≥7, respectively), and race (white vs black or other non-white race), we will then test whether this percentage differed by different group variable using independent samples *t* tests.

To further explore the trend in performance of all tasks (using devices for daily monitoring), we will compute proportions of missing data for each person over 2-week time periods for the entire 6-month follow up time for weight, Fitbit, and blood glucose. We will construct empirical summary plot of biweekly missingness. This consisted of plots of mean percentage missing at each biweekly period over 13 biweekly periods. At each time point, bars representing 1 SE below and above the mean are also included. To examine if the trends differ by important clinical group variables, these plots will also be produced by different age group, HbA_1c_ group, and race. We will fit a generalized linear model with this proportion as the dependent variable. Predictors included age and HbA_1c_ group, and race, with each predictor tested in separate models. Time will also be included to assess change in biweekly missingness over time, as well as interactions between time and each predictor, to assess differences in change in biweekly missingness over time by age and HbA_1c_ group and race. We will also test the change in perceived usefulness and ease of use of the devices by conducting a paired *t* test on these measures between baseline and 6-month follow-up survey.

We recognize that even though individuals may not provide data, they could still be compliant in weighing themselves, exercising, drawing blood glucose, and taking medication. The data may not transmit because (1) the device was not charged or (2) internet service was not available. Nevertheless, we will assess overall feasibility by examining data, which reflect both performing the measurement and being responsible for transmitting the data. Since in practice, this will be required for real-time monitoring.

#### Patient Interviews

A coding team comprised 4 pre- and postdoctoral students trained in qualitative analysis will code these qualitative data. We will analyze the transcribed interview data using directed content analysis [25]. We will create a codebook that describes the creation of inductive and deductive codes and themes. Codes will be developed and analyzed in the context of diabetes self-management, the use of mobile technologies to support self-management, and the data visualizations. We will use Microsoft Excel in the initial first-level coding process and then upload data into Atlas.ti version 8 (Berlin, Germany) to support higher level coding and analyses [26]. First, the coding team will independently read and code transcripts and then meet to discuss coding and emerging themes and reconcile coding differences. This process will continue until first-level coding is complete. Following the completion of first-level coding, we will begin to develop more refined codes and to identify patterns of data across all transcripts. The coding team will ensure reliability and validity throughout the process by meeting regularly to discuss code and theme development, creating a codebook with agreed upon definitions, and recording an audit trail of our actions.

#### Clinician Interviews

We will use the same process to code the transcribed clinician interviews. The themes and codes from the interviews will be mapped onto visual data trajectories developed from the mobile devices. We will then examine the data at particular points in time over the 6-month period to identify trends. The data will be described in relation to time of the year, demographics, and clinical characteristics of patients.

We will conduct interviews in which we will present data trajectories and interview themes and explore ways to address collaborative work for self-monitoring and addressing challenges in diabetes self-management. The analysis team, in collaboration with the study recruiter, will conduct a preliminary analysis of field notes and transcribed interviews. Text data will be analyzed as described above.

## Results

This study has received IRB approval. Enrollment of patients was completed in March 2018 with a sample size of 60 patients. At the patient level, data collection is complete, but data analysis is pending. At the clinician level, data collection is ongoing. Up to 20 health care providers will be enrolled for the clinician interviews.

## Discussion

### Overview

This study provides an overview of the methodology used in a study examining the feasibility of collecting near real-time data from mHealth technologies for patients with T2DM. Determining whether it is feasible for patients to use multiple mobile devices to self-manage their diabetes is an important first step in developing effective personalized care delivery strategies.

In addition to exploring the use of mobile technology to manage chronic illness, our approach has several strengths. First the use of mixed methods to address our aims is a strength of this study. The convergence of both quantitative and qualitative elements, data collection techniques, and analyses will facilitate a more in-depth and comprehensive approach to and understanding of diabetes self-management [27]. Another strength of our study is the use of data visualizations during participant interviews as an innovative approach to enhance discussion and gain insight into the challenges and successes participants experienced in diabetes self-management with mobile devices over the past 6 months. Presenting data this way could stimulate discussion and improve communication by graphically displaying patterns and trends [28-30].

We do acknowledge several limitations to this study. The first is the limited sample size, which does not allow us to draw statistical conclusions from the data. We are not testing the effect of any intervention, so cannot assess the impact on diabetes outcomes of using these technologies. Finally, we were not able to integrate these data into the EHR to assess pragmatic aspects of the use of the data at this time within the health system.

### Conclusions

Our study is among the first to seek answers to the many questions related to integrating patient data from mobile devices into diabetes self-management care delivery models and EHRs. Questions such as how long patients will track multiple types of diabetes-related data, which strategies will best help patients self-manage their diabetes using self-generated data, and how clinicians might effectively guide patients to better manage their disease in near real time need to be addressed in the emerging era of digital health.

## Abbreviations

API: application programming interface
eHealth: electronic health
EHR: electronic health record
HbA_1c_: hemoglobin A_1c_
IRB: institutional review board
mHealth: mobile health
REDCap: Research Electronic Data Capture
SMS: short message service
T2DM: type 2 diabetes mellitus
